# Polymer bilayer-Micro arc oxidation surface coating on pure magnesium for bone implantation

**DOI:** 10.1016/j.jot.2023.05.003

**Published:** 2023-05-24

**Authors:** Jieyang Dong, Jiaqi Zhong, Ruixia Hou, Xiaodong Hu, Yujiong Chen, Hangbin Weng, Zhewei Zhang, Botao Liu, Shengbing Yang, Zhaoxiang Peng

**Affiliations:** a Ningbo University Affiliated Li Huili Hospital, Ningbo University, Ningbo, 315040, China; b Ningbo University School of Medicine, Ningbo University, Ningbo, 315211, China; c Shanghai Key Laboratory of Orthopaedic Implants, Department of Orthopaedic Surgery, Shanghai Ninth People's Hospital, Shanghai Jiao Tong University School of Medicine, Shanghai, 200240, China

**Keywords:** Magnesium bone implant, Polymer coating, Micro-arc oxidation, Degradation control

## Abstract

**Background:**

Pure magnesium-based ortho-implants have a number of advantages. However, vital parameters like degradation rate and biocompatibility still call for significant improvement.

**Methods:**

In this study, poly (1,3-trimethylene carbonate) (PTMC) and polydopamine (PDA) bilayer and micro arc oxidation composite coatings were prepared successively on magnesium surface by immersion method and microarc oxidation. Its corrosion resistance and biocompatibility were evaluated by *in vitro* corrosion tests, cellular compatibility experiments, and *in vivo* animal experiments.

**Results:**

*In vitro* experiments demonstrated that the composite coating provides excellent corrosion protection and biocompatibility. Animal studies demonstrated that the composite coating slowed the degradation of the implant and was not toxic to animal viscera.

**Conclusion:**

In conclusion, the inorganic-organic composite coating proposed in this study provided good corrosion resistance and enhanced biocompatibility for pure magnesium implants.

**The translational potential of this article:**

The translational potential of this article is to develop an anti-corrosion composite coating on a pure magnesium surface and to verify the viability of its use in animal models. It is hoped to open up a new approach to the design of new degradable orthopedic magnesium-based implants.

## Graphical abstract

**Figure:**
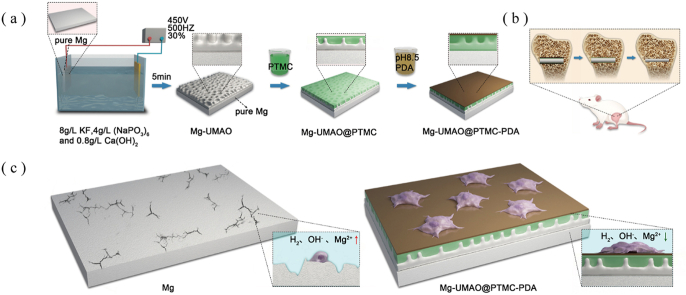

## 1 Introduction

The need for orthopedic equipment is gradually increasing as the older population grows. Permanent orthopedic implants made of bioinorganic metals including cobalt alloys, titanium alloys, and stainless steel have been utilized used to stabilize bones and restore function [1]. However, these bioinert materials have exerted several side effects such as bone density loss and osteoporosis due to the “stress shielding” effect [2]. And these non-degradable implants often require a second surgery to remove, increasing the health risks and medical costs for patients.

Magnesium (Mg) has multiple uses as an alternative to degradable orthopaedic implants. The Young's modulus of magnesium (40–45 ​GPa) is very close to the cortical bone of humans (10–30 ​GPa) compared to cobalt and titanium hence preventing the “stress shielding” effect [3]. In addition, Mg can disintegrate in a physiological environment completely while having excellent biocompatibility and osteoinductivity [4,5]. However, due to the fast degradation process of pure magnesium, the mechanical integrity and support properties of Mg implants could reduce rapidly, resulting in early fixation failure. Moreover, in local osseous tissue, the released magnesium ions and hydrogen are likely to accumulate during the rapid corrosion process, causing adverse tissue response and the formation of gas cavities [6]. Excessive hydrogen release has a negative impact on the survival rate of rats with implanted magnesium implants. In order to design magnesium implants that are more suitable for practical clinical applications, the issue of excessive gas cavities caused by degradation should be taken into serious consideration [7].

To improve the corrosion resistance of magnesium-based implants, many attempts have been introduced, such as surface modification treatment and alloying. However, these techniques have disadvantages when used alone. The use of magnesium alloys as orthopedic implants requires careful consideration of the biocompatibility issues arising from the release of ions from other alloying elements [[8], [9], [10], [11]]. And polymer coating could easily detach from the substrate since the connection was solely physical adhesion usually formed with the immersion method [12].To cope with the multiple challenges faced by magnesium-based orthopedic implants, we carried out a composite coating treatment on pure magnesium.

The advantage of pure magnesium over magnesium alloys is that it avoids the formation of secondary phases and the resulting galvanic corrosion, which ultimately leads to the rapid degradation of magnesium-based implants. Pure magnesium can also prevent involving toxic and harmful elements in the alloy from harming the human body [13]. Recent reports indicate that high-purity magnesium suture anchors exhibit appropriate degradation behavior, providing reliable anchoring functionality within 12 weeks of implantation in sheep, with no toxic effects on animal organs [14]. Pure magnesium screws used for anterior cruciate ligament (ACL) reconstruction have also been reported. Compared to the currently used poly (lactic acid) polymer screws in clinical practice, pure magnesium screws exhibit adequate initial mechanical performance during the proximal tibial fixation process [15].

As one of the anti-corrosion means of magnesium and its alloys, polymer coating technology can provide a physical barrier isolating the metal substrate from the physiological environment, thus retarding corrosion [[16], [17], [18]]. Polymers have a variety of physical and chemical properties that provide various options for different corrosion protection requirements [19,20]. In recent years, quite a few synthetic polymers have been used to prepare anticorrosive coatings, such as poly (lactic acid) (PLA), poly (1,3-trimethylene carbonate) (PTMC), and poly (ether imide) (PEI), etc. [[21], [22], [23], [24]]. These polymers can reduce the penetration of aqueous solutions to the surface of magnesium substrates and thus resist solution corrosion [17]. The application of this series of polymer coating technologies has brought great progress in the corrosion protection of magnesium metal.

PTMC coating can provide excellent corrosion protection for magnesium-based ortho-implants due to its inherent layer-by-layer degradation characteristics. Compared to bulk disintegrating type polymers, PTMC can retard the penetration of corrosive media and maintain the mechanical integrity of magnesium implants longer. However, the hydrophobicity and absence of active functional groups limited PTMC coating's further use [25].

The polydopamine coating inspired by mussel adhesive proteins has gained considerable attention for its easy fabrication and excellent coating properties [26]. Dopamine also exists naturally in the human body, such as in the nervous system [27]. By adding an alkaline buffer solution, dopamine can self-polymerize and form a thin and hydrophilic polymeric dopamine film on a wide range of material's surface [26]. Additionally, some studies have shown that PDA exhibits excellent adhesion properties, PDA can immobilize nucleophiles (e.g. amine and thiol groups) on its surface via Schiff base reactions and Michael addition reactions. It provides the possibility of subsequent surface modification for materials lacking active functional groups on the surface [28].

Regarding above mentioned properties, we combined PTMC and PDA coatings to not only improve the corrosion resistance of biodegradable pure magnesium ortho-implants, but also serve as a local drug delivery platform in the future, such as incorporating of growth factors or other drugs in polymer coatings to treat different orthopedic disease accordingly [[29], [30], [31], [32], [33], [34], [35], [36]].

However, the polymer coating can be separated from the substrate relatively easily because the connection between them is only a physical bond formed by the dipping method [37]. It's worth noting that because different materials have different chemical properties, a smooth substrate surface is usually not conducive to the adhesion of other materials, and a rough substrate surface can provide better adhesion for polymer coatings [38].

Therefore, we chose a common pretreatment method for magnesium and its alloys—ultrasonic micro arc oxidation (UMAO) to solve the above problems [39]. The UMAO film is composed of a dense inner film and an outer loose film. In order to achieve the corrosion resistance of magnesium and its alloys, it is crucial to increase the density and anchoring strength of the film [40,41]. Ultrasonic wave have been utilized to increase the movement of liquid particles and promote the metal oxides melting through cavitation [42], thereby thickening the dense inner film and enhancing the anchoring strength of the film [43]. The formation of the MgO ceramic layer by this simple, environmentally friendly and reproducible method can not only improve the bonding force between the polymer coating and the pure magnesium substrate, but also improve the corrosion resistance of the pure magnesium substrate.

In this work, we combined PTMC-PDA bilayer polymer with UMAO aimed at improving the corrosion resistance and enhancing the biocompatibility of pure magnesium substrate. PTMC-PDA bilayer polymer film sealed the blind holes of UMAO film, and rough UMAO layer provided a suitable surface for polymers to attach on. Finally, PDA treatment can impart active functional groups to the surface of the material, improving biocompatibility and broadening application scenarios for magnesium implants.

## 2 Materials and methods

### 2.1 Sample preparation

The pure magnesium (purity 99.98%) was purchased from China New Metal Materials Technology Co., Ltd, China. PTMC (Mw, 500 ​000 ​Da) was purchased from Jinan Daigang Biomaterial Co., Ltd, China. Dopamine hydrochloride and 1,4-dioxane was purchased from Aladdin Chemistry Co., Ltd, China. Other reagents including potassium fluoride (KF), hexametaphosphate ((NaPO_3_)_6_), calcium dihydroxide (Ca(OH)_2_) and anhydrous ethanol were purchased from Sinopharm Chemical Reagent Co., Ltd, China.

Pure magnesium was cut into 10.0 ​mm ​× ​10.0 ​mm ​× ​1.5 ​mm plates for *in vitro* tests and φ 2.0 ​× ​3.0 ​mm rods for implantation. Surfaces of the substrates were polished using silicon carbide emery paper with gradient grits of 800–2000 and ultrasonically treated with ethanol and distilled water. The samples were then dried at 60 ​°C under vacuum.

Power supply for micro arc oxidation was provided by Suzhou Kunruipeng Precision Mould Factory, China. Magnesium anode was treated with 30 ​kW bipolar pulse power with 450 ​V voltage, frequency of 500 ​Hz, and pulse width of 50 μs current for 5 ​min. The stainless steel cathode and magnesium anode were 50 ​mm apart. In the electrolyte, the main component was KF (8 ​g/L), followed by (NaPO_3_)_6_ (4 ​g/L) and Ca (OH)_2_ (0.8 ​g/L). The Mg-UMAO samples obtained after ultrasonic micro-arc oxidation treatment.

PTMC coating was prepared via immersion method. PTMC was dissolved in 1,4-dioxane to a final concentration of 33.33 ​mg/mL. Mg-UMAO samples was completely submerged in PTMC-dioxane solution and removed to form a polymer layer on the sample. The sample was air dried for 2 weeks under room temperature.

PDA coating was prepared according to previous report [26]. Each Mg-UMAO@PTMC sample was submerged in 100 ​ml of Tris–HCl buffer solution (pH ​= ​8.5) with 2 ​mg/mL dopamine for 5 ​h. The sample was rinsed with PBS and dried under room temperature.

### 2.2 Fabrication and characterization

The surface morphology of the samples and their cross-sections were observed through a field emission scanning electron microscope (FE-SEM, Hitachi SU-70, Japan). The elemental compositions of all the samples were determined by Energy Dispersive Spectroscopy (EDS). Coating adhesion was measured according to ASTM D3359-22. The bond strength between the substrate and its coating was measured by CSM NHT-2 Coating Adhesion Automatic Scratch Tester according to previous reports. An optical contact-angle meter (DSA100, Kruss, Germany) was used to measure static water contact angles, each sample was measured three times.

### 2.3 Electrochemical corrosion tests

The samples were immersed in PBS under RT for potentiodynamic polarization (PDP). The experiments were measured using an electrochemical workstation (CHI660E, ChenHua, China) at 1 ​mV ​s^−1^ between −2.0 and −1.0 ​V (compared to saturated calomel electrodes). Based on linear fitting and Tafel extrapolation, we interpolated corrosion potential (E_corr_) and corrosion current density (I_corr_). At a cathodic polarization section, the over potential was approximately 50 ​mV lower compared to the free corrosion potential.

### 2.4 *In vitro* corrosion tests

The corrosion resistance under aqueous environment of the test sample was evaluated by immersion in PBS solution. The pH value of the solution was detected with a pH meter (FE20, METTLER TOLEDO, Switzerland) on Day15 and Day30. The samples’ surface morphology after immersion tests was observed using FE-SEM.

### 2.5 Extraction of rat bone mesenchymal stem cells

Two-week-old SD rats (Vital River Laboratory Animal Technology, China) were used to extract rat bone mesenchymal stem cells (BMSCs). The medullary cavity of rat's femur and tibia were rinsed with MEM-α medium (Gibco, USA) containing 10% fetal bovine serum (Gibco, USA). BMSCs were cultured in standard conditions (37 ​°C, 95%/5% air/CO_2_) and seeded in complete media, which consisted of MEM-α containing 10% fetal bovine serum and 1% penicillin and streptomycin (Macklin, China). The culture medium was changed every 2 days. Cells were digested with 0.05% trypsin-ethylenediaminetetraacetic acid (EDTA) (Gibco, USA) for following experiments.

### 2.6 Cell morphology and adhesion

2.5 ​ml of cell suspension with a cell density of 5 ​× ​10^4^ ​cell/ml was added to each sample in a 6-well culture plate. TRITC-phalloidin (Solarbio, China) and DAPI (Solarbio, China) were used to stain the adhered cells. The cells were observed through a confocal microscope (TCS SP8, LEICA, Germany).

### 2.7 Cell viability

After sterilized with ultraviolet irradiation, Mg, Mg-UMAO, Mg-UMAO@PTMC, and Mg-UMAO@PTMC-PDA samples were placed in MEM-α medium to prepare for extracts. The specimen surface area to medium volume ratio was 1.25 ​cm^2^ ​mL^−1^. After 24 ​h, extracts were collected. Each 96-well culture plate was added with 100 ​μL of cell suspension to test the viability of the cells. After 24 ​h of cell attachment, the original medium was removed, replaced with extracts, and the cells were cultured for 7 days. On days 1, 3, and 7, cells were detected using the Cell Counting Kit-8 Kit (Bimake, China) according to the manufacturer's instructions.

### 2.8 Animal experiment

All surgical and animal care procedures and experimental protocols were reviewed and approved by the Animal Ethics Committee of Ningbo University and conducted under the guidelines of the Institutional Animal Care and Use Committee (approval number: NBU20220067). We used two-month-old Sprague–Dawley male rats (Vital River Laboratory Animal Technology, China) weighing ≈250 ​g. The animals were randomly divided into three groups with four animals in one group. Anesthesia was administered subcutaneously with pentobarbital sodium (80 ​mg/kg). To gain access to the medullary cavity, a hole (φ ​= ​2.0 ​mm) was drilled into the femoral stem. Rods with sizes of 3.0 ​mm ​× ​φ 2.0 ​mm ​Mg, Mg-UMAO, and Mg-UMAO@PTMC-PDA were implanted, respectively.

Four weeks after surgery, all animals were euthanized. Animals’ femurs were fixed in 10% paraformaldehyde (Aladdin, China) and assessed using micro-computed tomography (micro-CT). We acquired images at a resolution of 18 ​μm using a 40 ​kV tube potential, 250 uA intensity, and 240-ms integration time. Reconstructed images were used to determine the percentage of new bone formation. Based on the actual situation, the region of interest (ROI) was chosen from 2D imaging with a standardized threshold (>220) as mineralized tissue and a threshold (>184) as implants. CTAn was used to analyze micro-computed tomography (micro-CT) images to determine bone structure parameters, such as bone volume/tissue volume (BV/TV) and trabecular number (Tb). In the final step, CTVox was used to reconstruct 3D images of the samples. The mean and standard deviation were calculated using at least three replicates.

After completion of micro-CT imaging, bone tissue samples were mounted with 10% paraformaldehyde and embedded in resin (Technovit 7200 VLC, Kulzer, Germany). These samples were sliced and ground to a thickness of <50 ​μm using a slicing and grinding machine (EXAKT, Germany). Histological sections were stained with methylene blue acidic fuchsin and observed using a light microscope.

Other tissues were also collected, such as liver, kidney, heart, and lung. Paraformaldehyde was used to fix the animal tissues, followed by a series of alcohols that dehydrated them, and finally, paraffin was used to embed them. For histological analysis, 5 ​μm sections were stained with hematoxylin/eosin and imaged using a bright-field microscope (DM 1000, Leica, Germany).

### 2.9 Statistical analysis

Data collected were analyzed with GraphPad Prism 9. Differences between groups were analyzed and determined by one-way ANOVA followed by Tukey's post hoc test. P value less than 0.05 was considered significant.

## 3 Results and discussion

### 3.1 Fabrication and characterization

**Figure 1:**
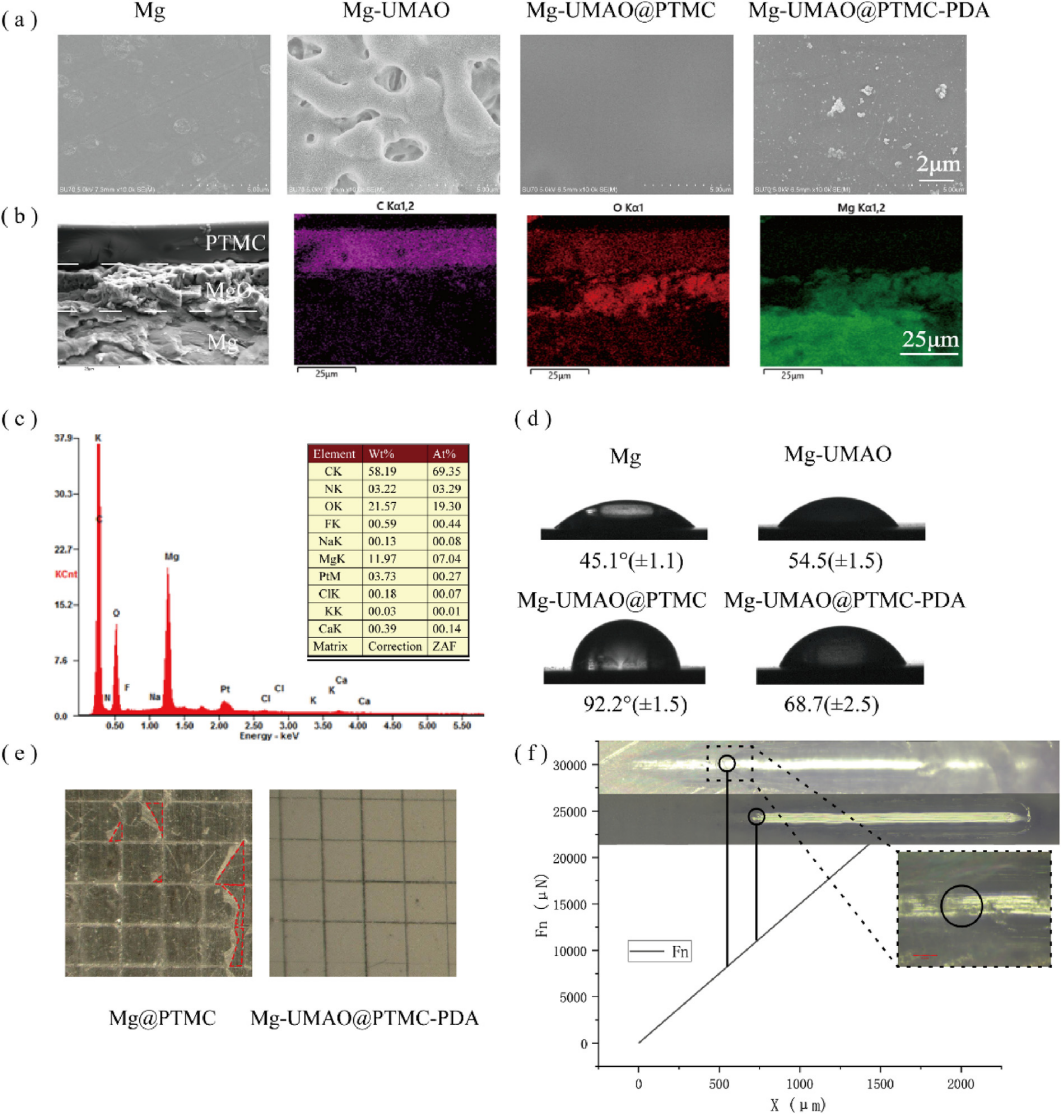
Characterization of Mg-UMAO@PTMC-PDA a) Scanning electron microscope photos after different surface treatments. b) Cross-sectional SEM and elemental mapping images of Mg-UMAO@PTMC-PDA. c) EDS spectrum and element content of Mg-UMAO@PTMC-PDA surface. d) Water contact angles after different coatings. e) Digital microscope images of Mg@PTMC and Mg-UMAO@PTMC after adhesion test (Area where PTMC coating was peeled off are marked red). f) Nanoscratch test results of Mg@PTMC and Mg-UMAO@PTMC. (For interpretation of the references to colour in this figure legend, the reader is referred to the Web version of this article.)

After the preparation of the pure magnesium surface composite coating, the microscopic morphology of the sample surface was observed with a scanning electron microscope (Fig. 1a).

Cross-sectional morphological SEM imaging, EDS and elemental mapping of Mg-UMAO@PTMC-PDA helped to reveal its structure (Fig. 1b). The thickness of inner UMAO layer and outer PTMC layer was 17.1 and 16.5 ​μm. As observed in SEM, UMAO layer exists blind holes with inconsistent sizes. During the UMAO process, the micro-arc discharge melts the surface of the magnesium substrate generating oxides and gases. These pores may be detrimental to the corrosion resistance of the UMAO coating, because the corrosive medium penetrates more easily into the magnesium substrate from here, and on the other hand increases the contact area between the corrosive medium and the UMAO coating. The PTMC seals the blind holes and forms a flatter surface for the UMAO@PTMC inorganic-organic composite coating, which could be observed in cross-sectional and superficial SEM images. After the composite coating was treated with polydopamine modification, the coating morphology did not change significantly with a few self-aggerated polydopamine particles deposited on the surface. The success deposition of PDA layer was further demonstrated with EDS elemental analysis performed on the sample surface (Fig. 1c). Nitrogen was detected with a content of 3.22%, indicating that PDA was deposited on the surface of PTMC.

Water contact angle measurements were also performed to assess the hydrophilicity of the samples (Fig. 1d). As shown, UMAO and PTMC increased the hydrophobicity, while the subsequent coating of PDA increased the hydrophilicity. Water contact angle measurements also demonstrated that the PDA coating was successfully prepared.

Adhesion force of the polymer coatings to the substrates was tested in accordance to ASTM D3359-22(Fig. 1e). The coating on Mg-UMAO@PTMC samples after removal of tape remained intact, while the coating on Mg@PTMC sample was partially peeled off, demonstrated UMAO improved the adhesion strength of the polymer coatings. Further quantitative adhesion force was measured using nanoindentation (Fig. 1f), the critical load for Mg-UMAO@PTMC is 11.0022 ​N, for Mg@PTMC is 8.24425 ​N. The adhesion force between polymer coating with the substrate is improved with the presence of UMAO layer.

### 3.2 *In vitro* degradation

To evaluate the degradation behavior of the four groups of samples, the generated OH^−^ (*i.e.,* pH) was measured every 15 days.

The degradation of magnesium-based implants in aqueous media is accompanied by the release of H_2_ and OH^−^, as shown in (a).

(a) Mg ​+ ​2H_2_O →Mg^2+^ ​+ ​H_2_ ​+ ​2OH^-^

**Figure 2:**
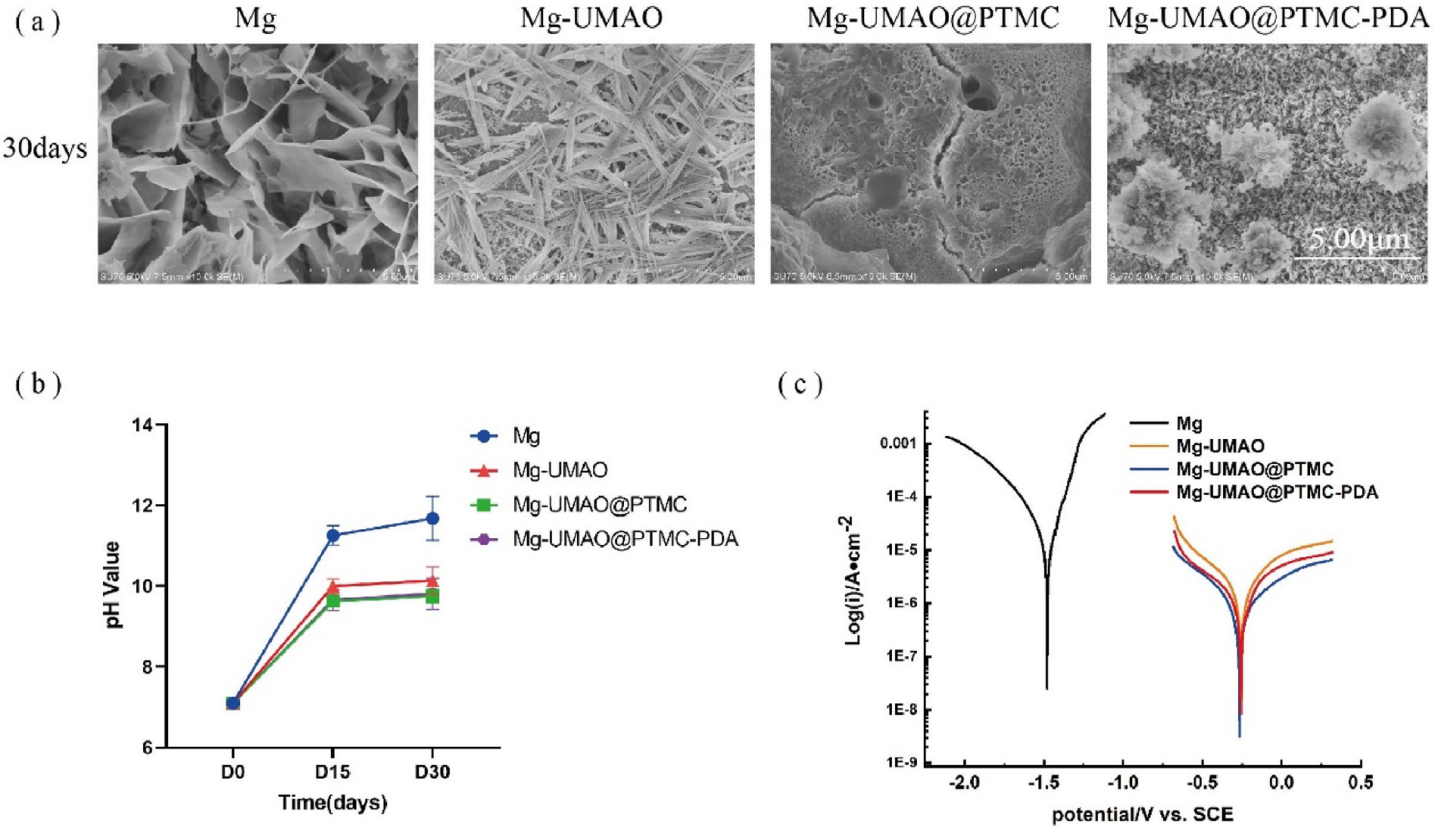
*In vitro* corrosion evaluation of Mg-UMAO@PTMC-PDA Pure Mg, Mg-UMAO, Mg-UMAO@PTMC, and Mg-UMAO@PTMC-PDA were immersed in PBS under room temperature. a) Scanning electron micrographs after 30 days of immersion. b) The pH changes of the immersed solution. c) The polarization curve of different samples.

To further study their degradation behavior, the surface morphologies of the four samples after immersion for 30 days were observed by scanning electron microscopy. After 30 days of immersion in PBS, severe corrosion could be observed in pure Mg group (Fig. 2a). Irregular holes and cracks formed on the substrate. UMAO coating protected Mg substrate from severe corrosion, however, the formation of crystals on the surface still indicates excessive degradation on the early stage.

Due to the presence of PTMC in UMAO@PTMC and UMAO@PTMC-PDA, the overall corrosion process would be slower. With no obvious crystals and much smaller cracks on the surface of samples, the degradation process is significantly slowed. In the UMAO@PTMC group, a small defect of PTMC was observed, and the defect exposed the structure of UMAO (Fig. 2a), contrary from the surrounding polymer exfoliation. We assume this is due to that PTMC layer couldn't remain intact at the end phase polymer degradation because the solution penetration and gas formation on the substrate. Polymer bilayer in the UMAO@PTMC-PDA remained complete and intact, indicating PTMC-PDA bilayer may exerts better corrosion resistance owing to PDA layer, however, we didn't carry out further investigation since we consider the protection of PTMC layer is sufficient to Mg substrate.

The solution pH after UMAO treatment rise was significantly slowed down, indicating that UMAO had a good protective effect on the magnesium substrate. Meanwhile, the pH of the solutions in the Mg-UMAO@PTMC and Mg-UMAO@PTMC-PDA groups decreased further (Fig. 2b).

Electrochemical testing was used to better understand the corrosion resistance of the four groups of samples (Fig. 2c). Tafel slope extrapolation were used to determine the free corrosion potential E_corr_ and corrosion current density I_corr_. Compared with Mg, the E_corr_ of Mg-UMAO raised from −1.48 to −0.25 ​V (vs SCE), reducing the thermodynamic tendency of galvanic corrosion. Furthermore, the I_corr_ of Mg-UMAO was 1.59 uA∙cm2, which was much lower than that of Mg (24.0 uA∙cm2) (Table 1). It showed that the corrosion rate was significantly reduced after UMAO treatment. The addition of PTMC further enhanced the corrosion resistance (E_corr_ ​= ​-0.26 ​V, I_corr_ ​= ​0.43 uA∙cm2), and the addition of PDA did not alter the overall parameters significantly (E_corr_ ​= ​-0.26 ​V, I_corr_ ​= ​0.58 uA∙cm2).

**Table 1:** Electrochemical parameters for pure Mg, Mg-UMAO, Mg-UMAO@PTMC, and Mg-UMAO@PTMC-PDA in PBS solution.

Sample	Icorr (uA∙cm2)	E_corr_(V)	CR
Mg	24.019	−1.4781	0.28252
Mg-UMAO	1.5888	−0.24934	0.018688
Mg-UMAO@PTMC	0.42507	−0.25737	0.0049998
Mg-UMAO@PTMC-PDA	0.58284	−0.26185	0.0068555

According to the above results, Mg-UMAO@PTMC and Mg-UMAO@PTMC-PDA exhibited the best *in vitro* corrosion resistance among the four groups of samples.

Magnesium-based implants would gradually corrode in the physiological environment after implantation. The anti-corrosion mechanism of Mg-UMAO@PTMC-PDA was proposed in Fig. 1c. As shown, all components of the composite coating affected the biodegradation behavior of Mg substrates. The MgO ceramic structure in the innermost layer provided a rough surface, which provided good conditions for the adhesion of PTMC. Unlike the overall disintegration mode of poly (lactic acid) (PLA), poly (lactic-co-glycolic acid) (PLGA), or polycaprolactone (PCL), PTMC followed a layer-by-layer degradation mode, which helped to slow down the corrosion rate of magnesium-based materials. Therefore, this composite coating could effectively prevent the rapid degradation of magnesium-based implants and maintain their integrity and mechanical properties in the early stage of implantation.

### 3.3 Cytocompatibility evaluation

**Figure 3:**
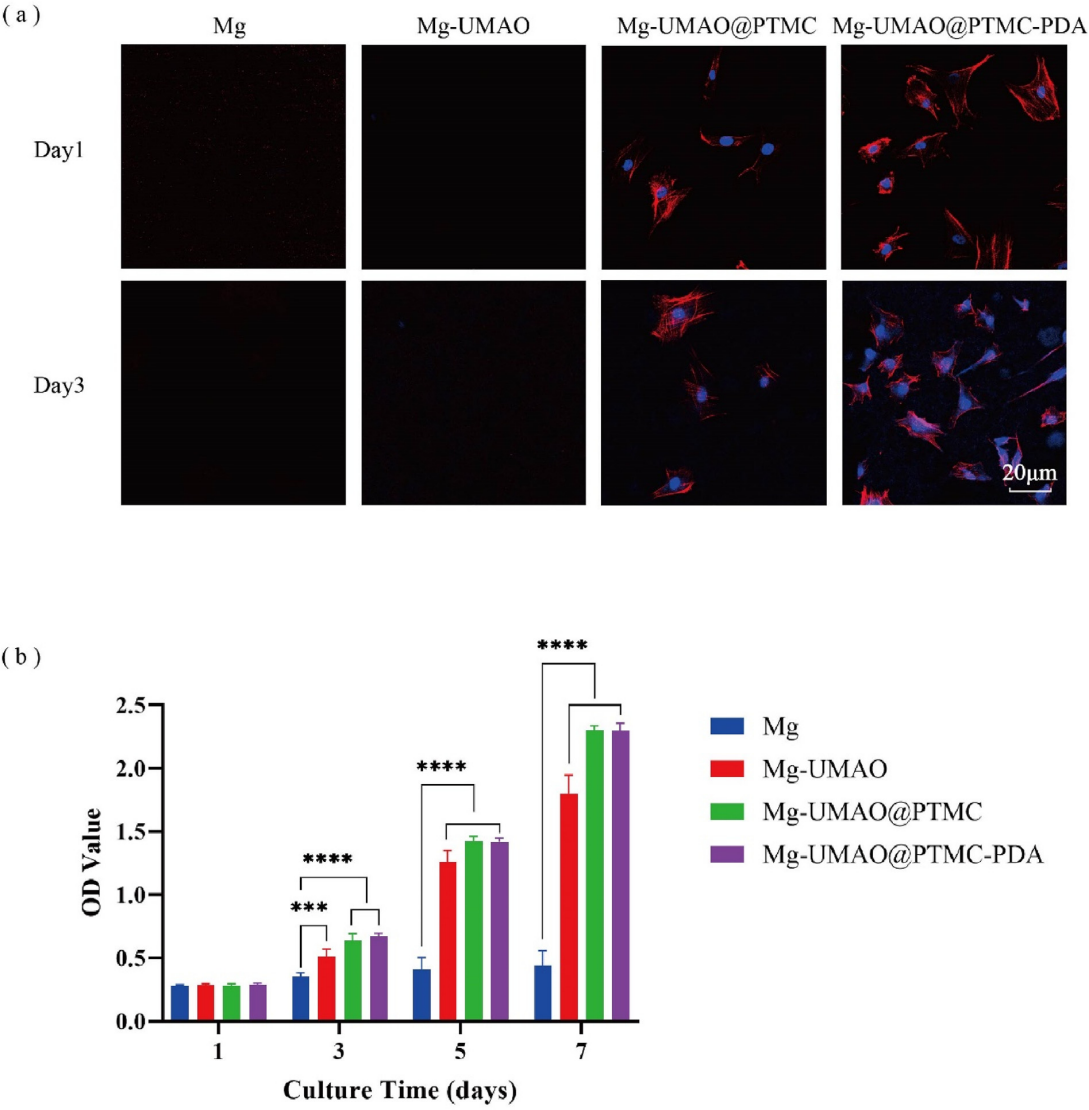
Biocompatibility of Mg-UMAO@PTMC-PDA a) Confocal images of adhered BMSCs on pure Mg, Mg-UMAO, Mg-UMAO@PTMC, and Mg-UMAO@PTMC-PDA, cells were stained with TRITC-phalloidin (Red channel) and DAPI(blue channel). b) Proliferation of BMSCs cultured with sample extracts, cell viability measured by CCK-8. ∗∗∗P＜0.0005, ∗∗∗∗P＜0.0001. (For interpretation of the references to colour in this figure legend, the reader is referred to the Web version of this article.)

Biocompatibility is crucial for implant materials as the implant interacts with the surrounding tissue [[44], [45], [46], [47], [48]]. Implants enhancing cell adhesion and proliferation could significantly accelerate the bone integration as implant gradually degrades. Therefore, we assessed cell adhesion on the surfaces of Mg, Mg-UMAO, Mg-UMAO@PTMC, and Mg-UMAO@PTMC-PDA samples and their biocompatibility in the extracts. The cytoskeleton was stained with TRITC-phalloidin (red), and nuclei were stained with DAPI (blue). No viable cells were observed on Mg and Mg-UMAO samples due to excessive degradation rate. The surface morphology was not favorable for cell adhesion as well (Fig. 3a).

Viable cells could be observed on Mg-UMAO@PTMC samples, the degradation is controlled hence cells could attach to the surface of the sample and adhere to it. However, its inherent hydrophobicity was not suitable for cell adhesion. After PDA treatment, the surface of the Mg-UMAO@PTMC-PDA group became suitable for cell adhesion and spreading, with better expression of filamentous actin and larger cell area. Since the surface morphology was not significantly changed after PDA modification, the hydrophilic surface and reactive groups provided by the PDA layer play a key role in promoting cell adhesion and proliferation.

It is well known that for medical implants, the interaction between the implant and the surrounding tissue is crucial. Mammalian cells cultured on substrates typically undergo adhesion, spreading, cytoskeleton development, survival and proliferation [49]. A material surface that helps cells adhere well can promote cell proliferation.

The role of the microenvironment around degradable implants on cell proliferation is also important for osseointegration between the implant and the tissue. Fig. 3b shows the cell viability of BMSCs cultured in the Mg, Mg-UMAO, Mg-UMAO@PTMC and Mg-UMAO@PTMC-PDA groups of extracts at 1, 3, 5 and 7 days. There was no significant difference in the proliferation of cells in each group of extracts after 1 day. After 3, 5 and 7 days of incubation, the viability of BMSCs cultured in the extracts of the three anti-corrosion-treated materials was higher than the pure Mg group. The results indicated that rapid degradation of magnesium-based implants resulted in a microenvironment around the implants that was not conducive to cell survival. With the extension of culture time, the UMAO group gradually showed the effect of improving biocompatibility by slowing down the degradation of magnesium-based implants, and the cell proliferation was significantly better than that of the pure magnesium group. The addition of PTMC helped seal the pores of UMAO and further improved the anti-corrosion properties of the coating, resulting in improved biocompatibility. The PDA coating did not have a significant effect on promoting cell proliferation in the leachate experiment. The above results suggested that Mg-UMAO@PTMC and Mg-UMAO@PTMC-PDA have better cytocompatibility, which may be related to the slower degradation of magnesium-based implants and less pH change in the microenvironment. Due to the tight bond between UMAO and PTMC coating, the corrosion medium could not reach the inorganic magnesium part of the implant before it penetrates the composite coating. Considering the good cytocompatibility of both the surface and the extract of the Mg-UMAO@PTMC-PDA implant, it was expected to be used for *in vivo* bone repair.

### 3.4 Analysis of *in vivo* bone response and degradation

To evaluate the *in vivo* anticorrosion performance and biocompatibility, we fabricated Mg, Mg-UMAO, and Mg-UMAO@PTMC-PDA rods and implanted them into rat femoral defect sites (Fig. S1). Four weeks after surgery, the rats were sacrificed, and micro-CT was performed immediately to assess the degree of degradation of the magnesium-based implants and to examine the healing of the surrounding bone tissue.

**Figure 4:**
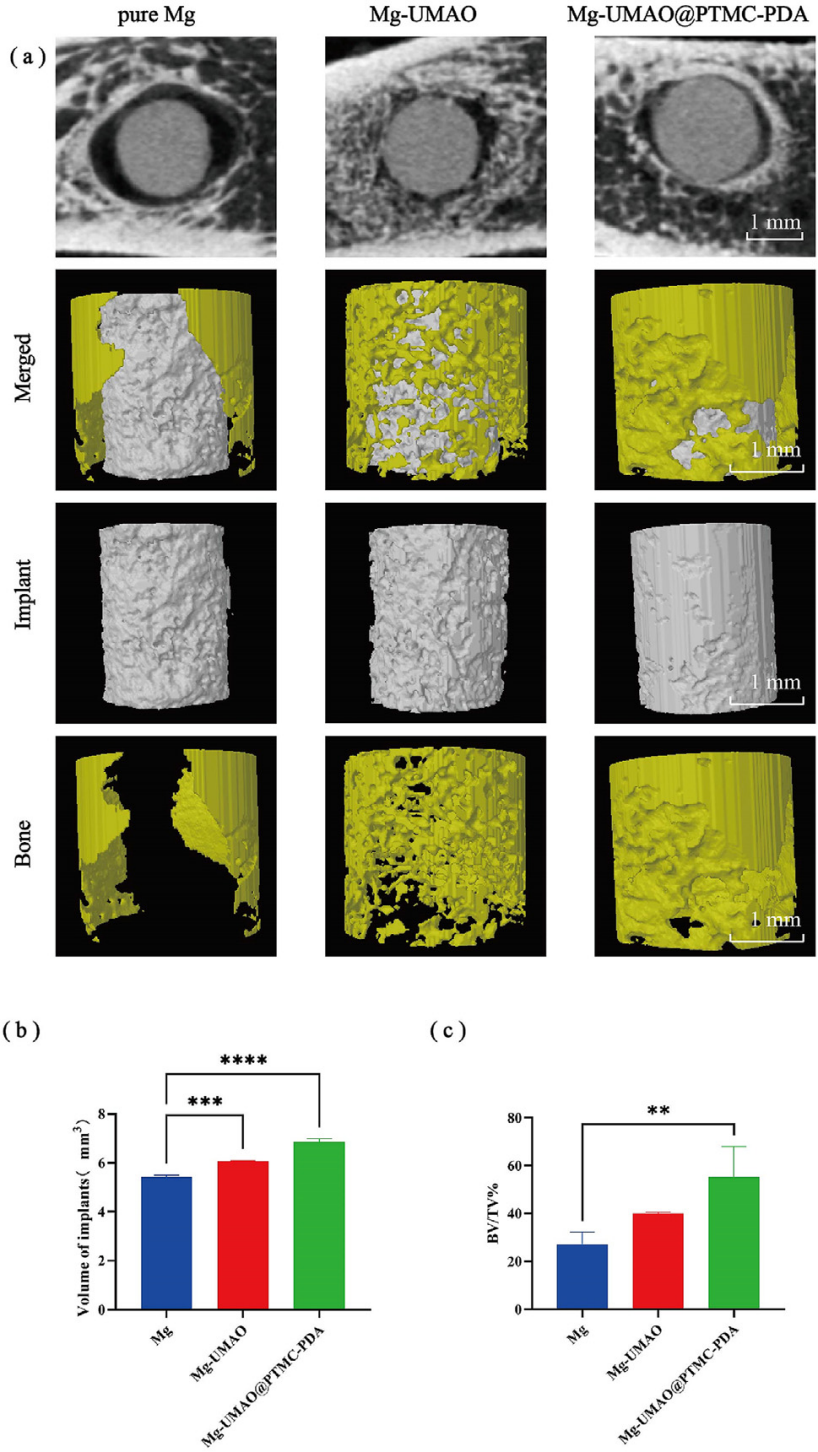
*In vivo* degradation evaluation via bone defect model Radiographs of implants in rat femur 4-weeks after implantation. a) 3D reconstruction of cortical bone and implants. b) Changes in implant volume. c) Bone volume fraction (BV/TV%). ∗∗P＜0.01,∗∗∗P＜0.001, ∗∗∗∗P＜0.0001.

Corrosion was evident in the Mg group (Fig. 4a). Mg group has the greatest decrease in volume, moreover, apparent peri-implant air cavities can be observed in the Mg group. In contrast, the surface corrosion on the other two groups with anti-corrosion treatment was milder pure Mg. It was worth noting that the Mg-UMAO@PTMC-PDA group had the best retention of implant morphology and closer contact with the surrounding bone tissue. After 4 weeks of implantation, the implants in the Mg-UMAO@PTMC-PDA group were more volumetrically preserved and more structurally intact than pure Mg rod (Fig. 4b). Aside from their excellent biocompatibility and anticorrosion properties, Mg-UMAO@PTMC-PDA also appeared to have good osteoinductivity. Bone growth was observed around the Mg-UMAO@PTMC-PDA. 3D images and qualitative analysis showed that the bone volume fraction (BV/TV) was higher in the Mg-UMAO@PTMC-PDA group than in the Mg group (Fig. 4c). Previous studies had shown that magnesium-based implants degrade rapidly shortly after implantation, producing large amounts of hydrogen gas and causing an increase in the pH of the local microenvironment, which is detrimental to normal cellular activity [[50], [51], [52], [53], [54]]. In combination, UMAO@PTMC-PDA coatings protected the implant from rapid biodegradation and attaches well to the surrounding bone tissue.

**Figure 5:**
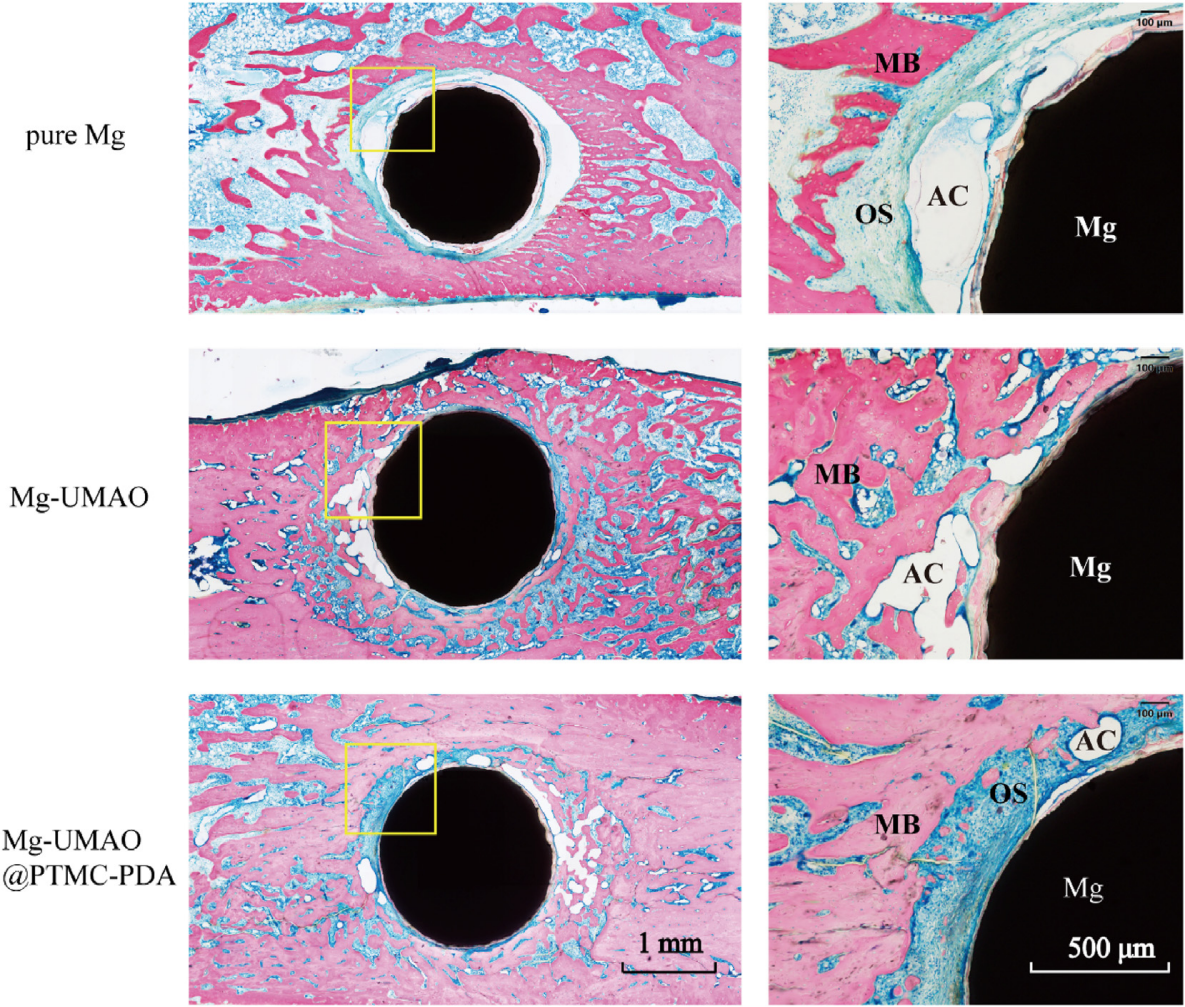
Histological evaluation of implant-bone interface Representative histological images of magnesium-based implants, the magnified region is marked by yellow rectangle, sections stained with methylene blue acid fuchsin (MB, mineralized bone; OS, osteoid; AC, air cavity; Mg, Magnesium implant). (For interpretation of the references to colour in this figure legend, the reader is referred to the Web version of this article.)

Histological images of Mg, Mg-UMAO, and Mg-UMAO@PTMC-PDA implants were shown in Fig. 5, and sections were stained with methylene blue-acidic fuchsin staining solution. Osteoblasts and osteoclasts were indicated in blue, osteoid were indicated in blue-green, newly mineralized bone were indicated in dark red, mature bone were indicated in brick red, and uncorroded magnesium implants were in black. The implants in the Mg group showed severe surface morphological erosion and significant air cavities were observed around the implants, which could explain the complete separation of the implant surface from the bone trabeculae. The Mg group had poor biocompatibility of the implant due to intense corrosion, resulting in less active osteoblasts and osteoclasts around the implant than the Mg-UMAO and Mg-UMAO@PTMC-PDA groups. In the Mg-UMAO and Mg-UMAO@PTMC-PDA groups of implants, a slowing down of the corrosion rate and a significant reduction of the air cavity around the implant could be observed in both groups. It was worth noting that the Mg-UMAO@PTMC-PDA group implants maintained the best shape with an almost complete circle in the cross-section.

In the construction of this composite coating on magnesium implants, the PTMC polymer acted as an intermediate layer, which was long-term stable and highly biocompatible. The biodegradation of the PTMC coating minimized substrate exposure and allowed the implant to last longer in the body and maintain a lower rate of degradation. The slow release of these degradation products could greatly accelerate bone formation [[55], [56], [57]]. Due to the multiple protection of the composite coating, the rapid local corrosion of magnesium-based implants in the early stage of the body could be avoided, which was beneficial to maintain the mechanical properties of the implants.

**Figure 6:**
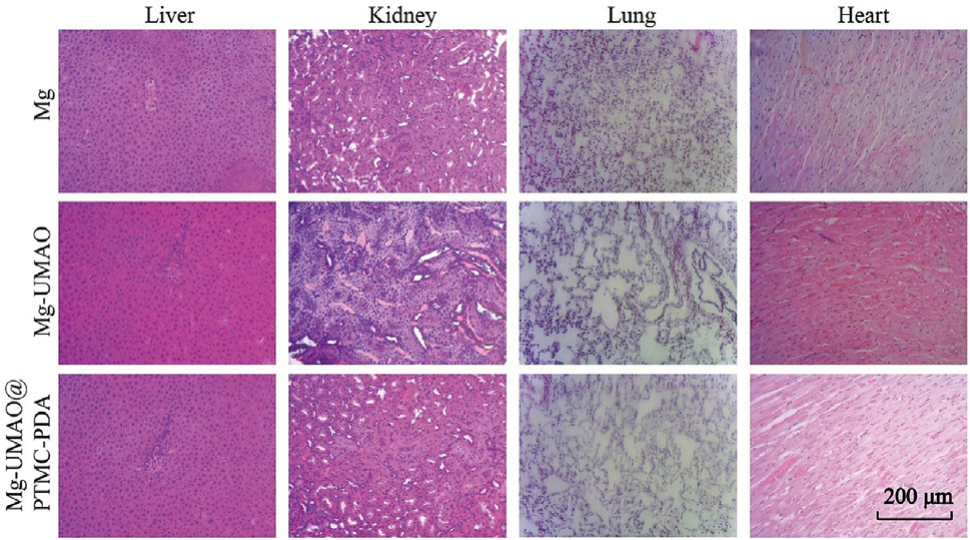
Histological evaluation of implant biocompatibility histological images of muscle, liver, kidney and heart 4 weeks after implantation.

After 4 weeks, no histological abnormalities were found in the livers, kidneys, lungs, and hearts of the 4 groups of mice, indicating that the magnesium-based implants and their degradation products have good biocompatibility *in vivo* (Fig. 6).

## 4 Conclusion

In this study, a composite coating consisting of UMAO, PTMC, and PDA was designed to slow down the degradation rate of magnesium-based implants and to meet clinical applications. SEM analysis showed the successful preparation of each coating, as well as the successful sealing of UMAO by PTMC. The surface hydrophilicity of the material was improved due to the modification of PDA. Cell adhesion and proliferation experiments showed that UMAO significantly enhanced the biocompatibility of the leachate, and the modification of PTMC and PDA facilitated cell adhesion and proliferation. *In vivo* experiments showed that Mg-UMAO@PTMC-PDA implants were less degraded than untreated pure Mg implants and were more tightly adherent to surrounding tissues in the implants.

Overall, our study provides a good strategy for the development of biodegradable magnesium-based orthopedic implants with good biocompatibility and promises to expand their application in drug loading.

## Credit author statement

Category 1:Conception and design of study: Jieyang Dong, Ruixia Hou, Zhaoxiang Peng. acquisition of data: Jieyang Dong, Jiaqi Zhong, Xiaodong Hu, Yujiong Chen, Hangbin Weng, Zhewei Zhang, Botao Liu. analysis and/or interpretation of data: Jieyang Dong, Jiaqi Zhong.Category 2:Drafting the manuscript: Jieyang Dong, Jiaqi Zhong. revising the manuscript critically for important intellectual content: Shengbing Yang, Zhaoxiang Peng.Category 3:Approval of the version of the manuscript to be published (the names of all authors must be listed):Jieyang Dong, Jiaqi Zhong, Ruixia Hou, Xiaodong Hu, Yujiong Chen, Hangbin Weng, Zhewei Zhang, Botao Liu, Shengbing Yang, Zhaoxiang Peng.

## Section I

The authors whose names are listed immediately below certify that they have NO affiliations with or involvement in any organization or entity with any financial interest (such as honoraria; educational grants; participation in speakers’ bureaus; membership, employment, consultancies, stock ownership, or other equity interest; and expert testimony or patent-licensing arrangements), or non-financial interest (such as personal or professional relationships, affiliations, knowledge or beliefs) in the subject matter or materials discussed in this manuscript.

## Declaration of competing interest

A conflict of interest occurs when an individual's objectivity is potentially compromised by a desire for financial gain, prominence, professional advancement or a successful outcome. The Editors of the Journal of Orthopaedic Translation strive to ensure that what is published in the Journal is as balanced, objective and evidence-based as possible. Since it can be difficult to distinguish between an actual conflict of interest and a perceived conflict of interest, the Journal requires authors to disclose all and any potential conflicts of interest.
