# Supplementary material for: Polymer bilayer-Micro arc oxidation surface coating on pure magnesium for bone implantation
Source: J Orthop Translat. 2023 May 24;40:27–36. doi: 10.1016/j.jot.2023.05.003 (PMC10232471; doi:10.1016/j.jot.2023.05.003)
Supplement: Multimedia component 1 [file mmc1.docx]

Supplementary material for

**Polymer bilayer-Micro arc oxidation surface coating on pure magnesium for bone implantation**

Jieyang Dong, Jiaqi Zhong, et al.


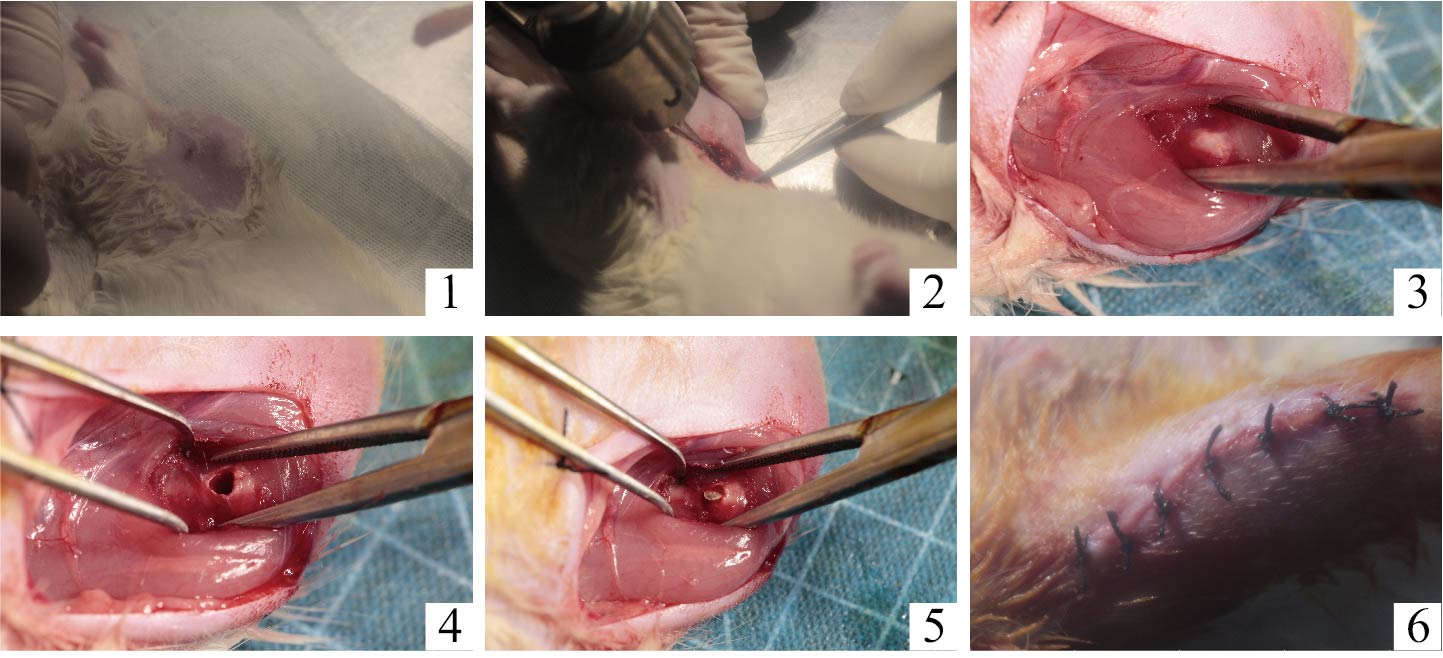


Figure S1

Rat femur surgery procedure.
